# Metastatic Intestinal Adenocarcinoma: A Rare Case Involving the Mandible

**DOI:** 10.1007/s12105-024-01697-4

**Published:** 2024-09-30

**Authors:** Baishakhi Modak, Chetana Chandrashekar, Adarsh Kudva, Raghu Radhakrishnan

**Affiliations:** 1 Department of Oral and Maxillofacial Pathology, Manipal College of Dental Sciences, Manipal, Manipal Academy of Higher Education, Manipal, Karnataka India; 2 Department of Oral and Maxillofacial Surgery, Manipal College of Dental Sciences, Manipal, Manipal Academy of Higher Education, Manipal, Karnataka India; 3 Academic Unit of Oral and Maxillofacial Medicine and Pathology, School of Clinical Dentistry, University of Sheffield, Sheffield, S10 2TA UK

**Keywords:** Intestinal-type adenocarcinoma, Adenocarcinoma, Metastasis, CK20

## Abstract

**Background:**

Metastatic intestinal adenocarcinoma involving the mandible is rare, posing diagnostic challenges because of its unusual presentation.

**Case presentation:**

A 55-year-old male presented with a rapidly growing mass in the right mandible, accompanied by facial asymmetry and vestibular obliteration. Histopathological examinations revealed features consistent with adenocarcinoma. Immunohistochemical analysis supported the diagnosis of intestinal adenocarcinoma, with subsequent metastasis confirmed by PET scan findings.

**Diagnosis:**

The lesion was conclusively diagnosed as intestinal adenocarcinoma metastasizing to the mandible.

**Management:**

The patient pursued treatment at a government facility, leading to a loss of follow-up.

A 55-year-old male was referred with a 20-day history of growth in the right lower jaw following the extraction of right mandibular second molar. On intraoral examination, a proliferative mass was observed on the residual alveoli, accompanied by vestibular obliteration (Fig. 1). An orthopantomogram (OPG) revealed diffuse radiolucency adjacent to tooth 47 (equivalent to tooth 31 by ADA numbering) and an altered bony pattern (Fig. 2). Given the rapidly expanding mass, the clinical diagnosis was a minor salivary gland malignancy, likely primary adenocarcinoma.

**Fig. 1:**
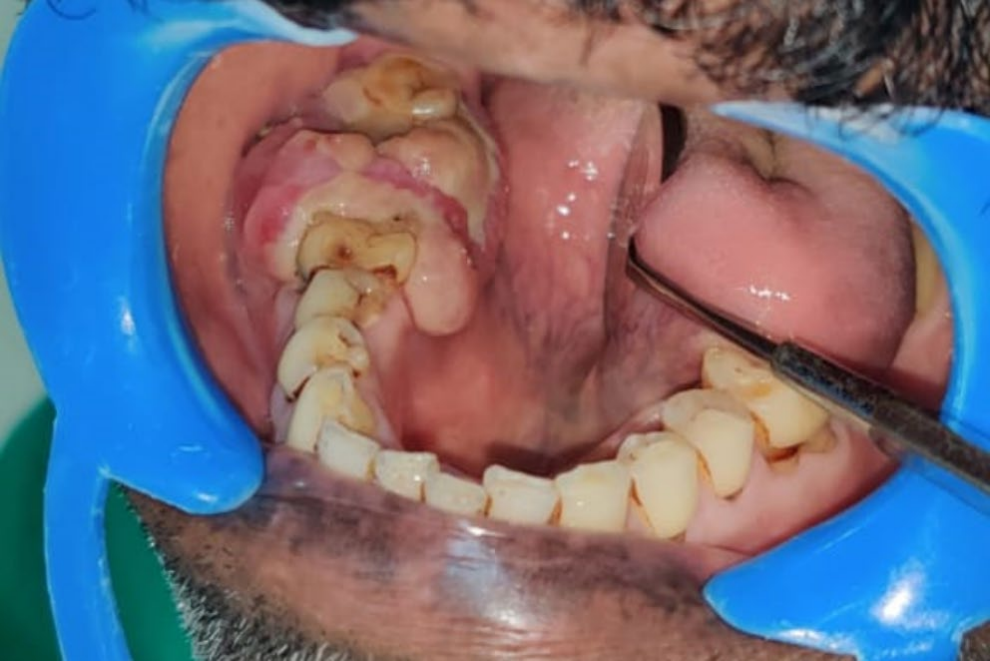
A proliferative mass on the residual alveolar ridge of the right mandibular arch, with vestibular obliteration

**Fig. 2:**
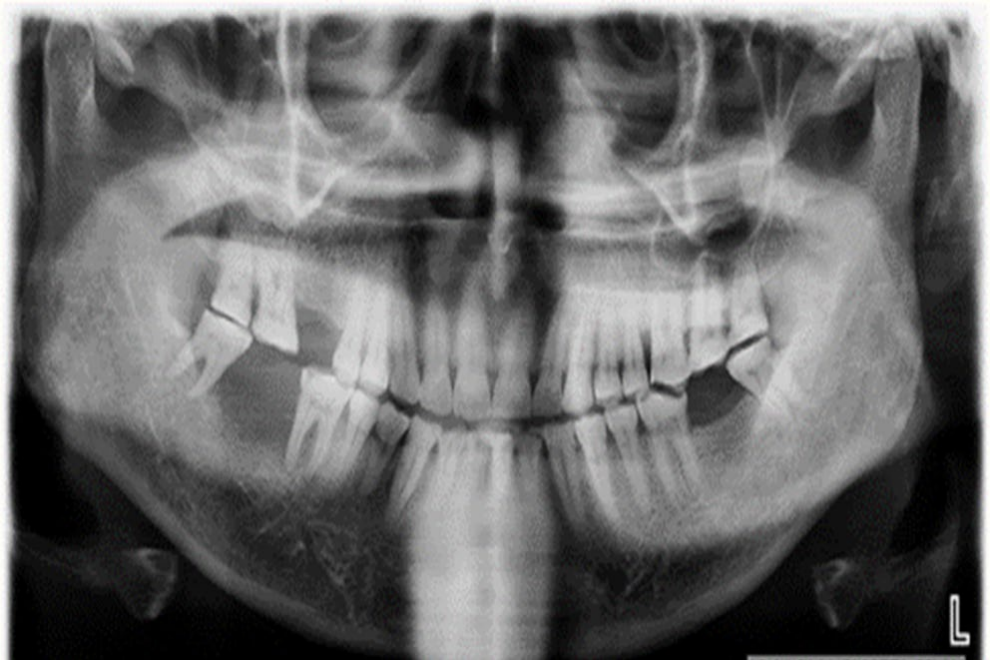
Orthopantomogram showing diffuse radiolucency in the edentulous area adjacent to tooth 47

The contrast-enhanced computed tomography from the patient’s medical records revealed heterogeneously enhancing circumferential wall thickening in the upper rectum and rectosigmoid regions, along with perilesional fat stranding, extramural extension, and infiltration into the presacral space and urinary bladder, consistent with malignancy. Additionally, multiple metastatic perirectal and pelvic lymph nodes (6–8 in number) were noted, corresponding to a T4bN2aM0 classification, indicating Stage IIIc disease.

An incisional biopsy was performed under local anaesthesia to arrive at a definitive diagnosis. Microscopic examination revealed an unremarkable stratified squamous epithelium without any evidence of dysplasia. The subepithelial tissue revealed cystically dilated irregularly shaped glandular tissue with varying patterns of differentiation and cytological atypia suggestive of adenocarcinoma. Goblet cells and Paneth cells were present in variable proportions. Although cellular atypia was not remarkable, the nuclei were hyperchromatic and had a cigar-shaped appearance (Fig. 3). Immunohistochemical examination revealed positive cytoplasmic staining for CK20 (Fig. 4), whereas HER-2NEU and CK7 exhibited negative membranous staining.

**Fig. 3:**
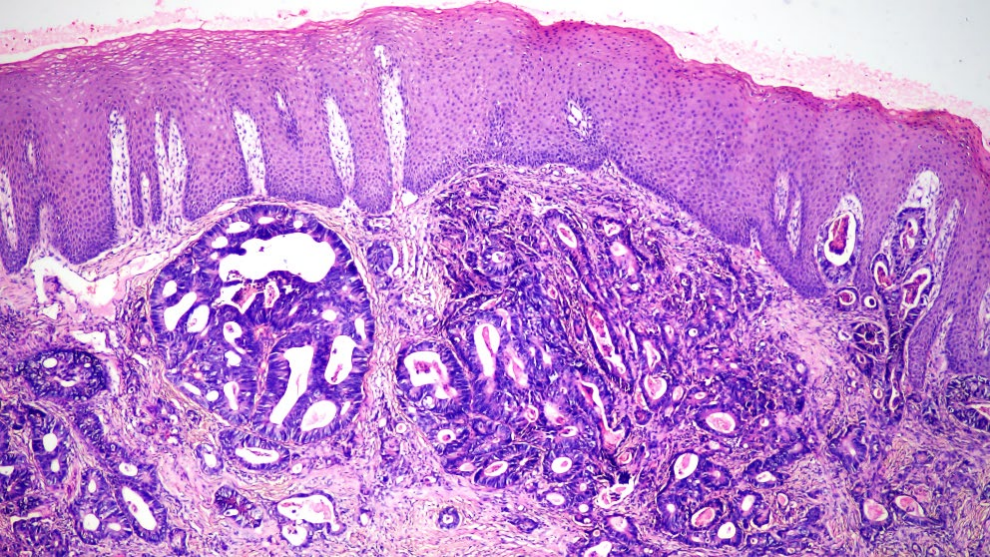
Microscopic examination showing features of adenocarcinoma with a mixed tubular and papillary growth pattern and the presence of goblet and Paneth cells. (H and E, 4x)

**Fig. 4:**
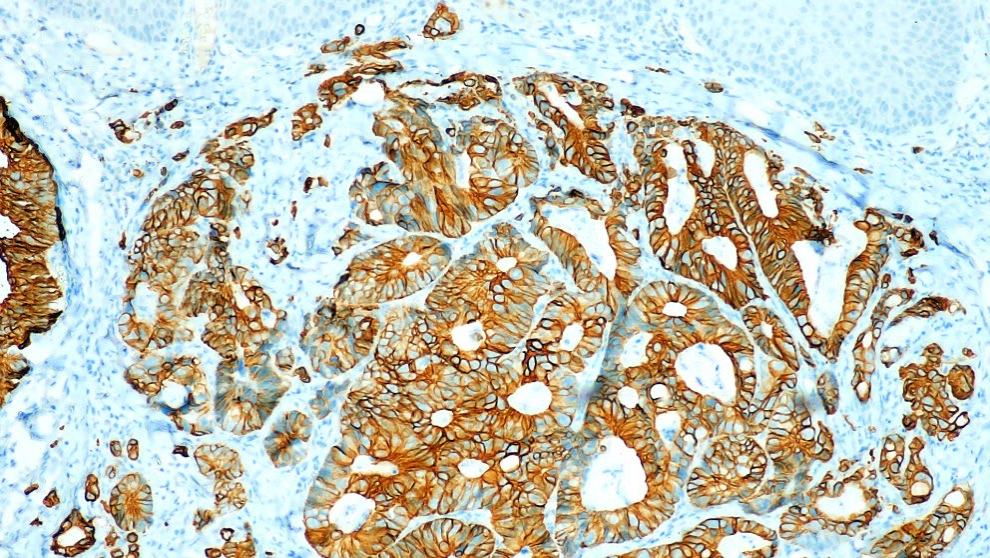
Tumor cells show positive cytoplasmic staining for CK20 by immunohistochemistry (CK20, 10x)

The recent FDG PET/CT scan revealed significant disease progression compared to the previous scan. Findings included the interval development of multiple hypermetabolic soft tissue lesions in the perirectal bed, indicating recurrence. Additionally, there was an increase in size and metabolic activity of several new bilateral lung nodules, newly enlarged mediastinal lymph nodes (Fig. 5), and hypermetabolic sclerotic lesions at previously affected skeletal sites (Fig. 6), all consistent with metastasis.

**Fig. 5:**
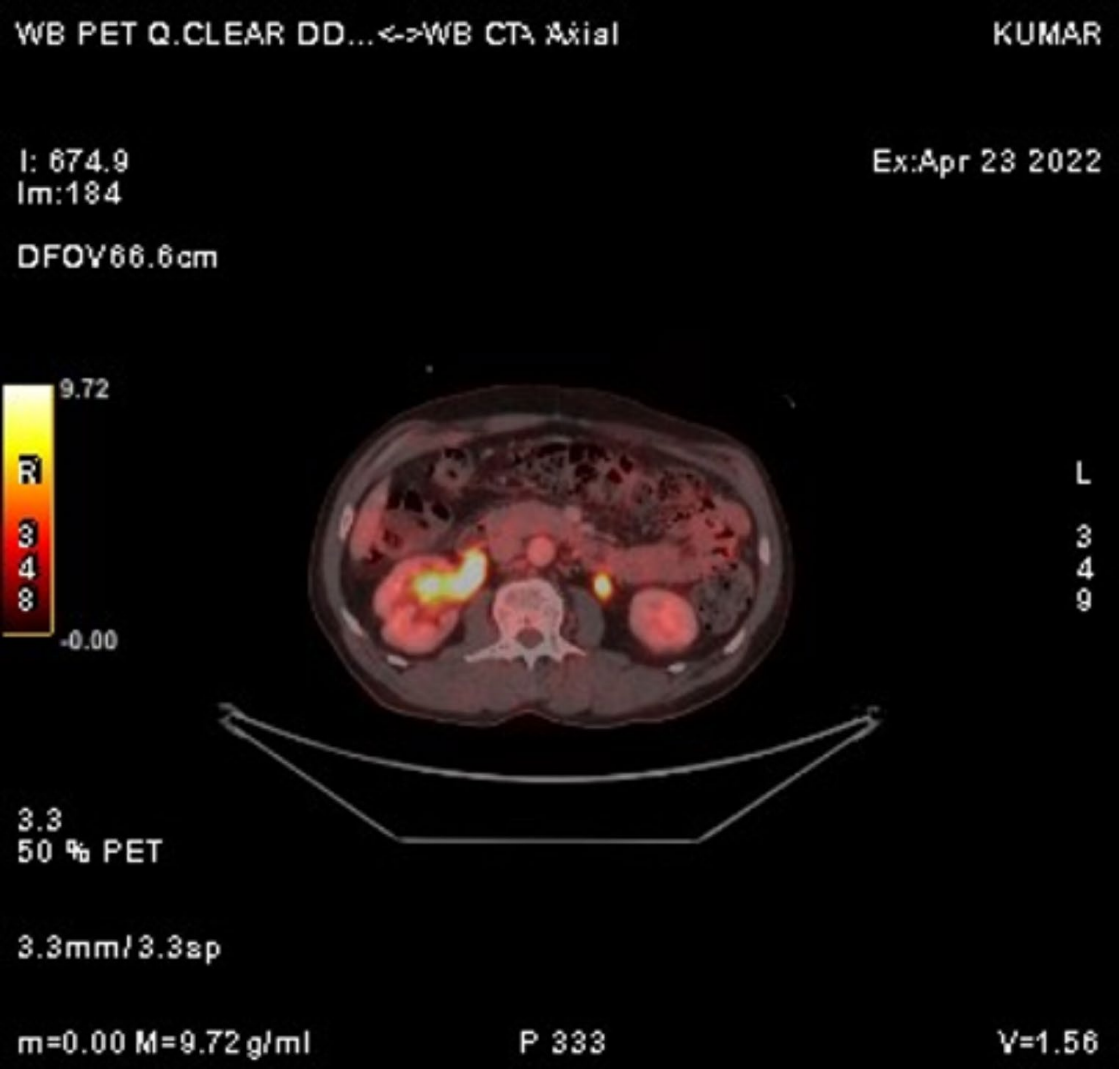
FDG PET/CT showing progression with hypermetabolic lesions and the development of bilateral lung nodules and mediastinal lymphnodes

**Fig. 6:**
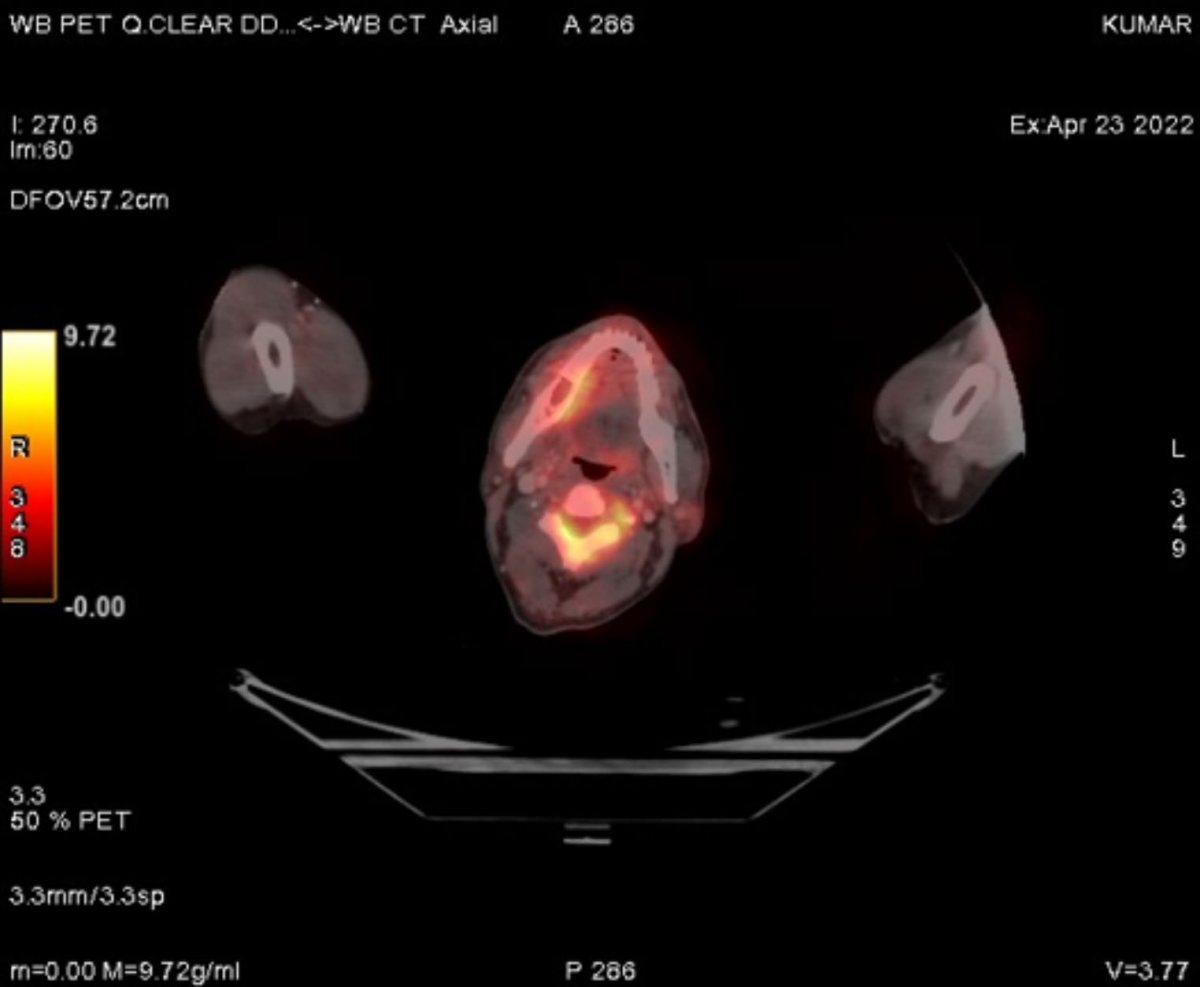
FDG PET CT scan showing a hypermetabolic lesion in the mandible, consistent with metastatic involvement

Following a comprehensive assessment, the final diagnosis was intestinal-type adenocarcinoma (ITAC) with metastasis to the mandible. The patient, constrained by financial assistance, opted for palliative treatment at a government facility, resulting in a loss to follow-up.

ITAC are aggressive cancers that often spread to the orbit, base of the skull, and intracranial space but rarely metastasize to the mandible. Histologically, there is an observable glandular epithelium lined with columnar epithelial cells, accompanied by Paneth cells and goblet cells. Immunohistochemically, ITAC demonstrate positive cytoplasmic staining for CK20 [1]. For the diagnosis of metastatic ITAC of the oral cavity, it is crucial to first exclude primary adenocarcinoma originating from the salivary gland. When CK7 immunostaining is absent and the sample lacks nonneoplastic small salivary glands adjacent to the tumour, identifying ITAC originating from minor salivary glands becomes particularly challenging [2, 3]. 

Metastatic dissemination affecting the jaw bones and soft tissues of the oral cavity can result from diverse primary neoplasms. The most common origin of carcinoma resulting in metastasis to the jaw bones is breast carcinoma in women and lung carcinoma in men. Metastatic spread may occur via the hematogenous route, transcoelemic permeation, the lymphatic route, local infiltration, or a combination of these mechanisms. Typically, the posterior part of the mandible, which is rich in marrow space, is often targeted for metastasis through the hematogenous route [4]. Upon detection of these metastatic lesions, determining the primary malignant site through diagnosis is crucial.

Early detection and accurate diagnosis are crucial for managing metastatic ITAC involving the mandible, emphasizing the importance of an interdisciplinary approach and advanced diagnostic modalities for optimizing patient outcomes.

## Data Availability

No datasets were generated or analysed during the current study.
